# Real-time inference for binary neutron star mergers using machine learning

**DOI:** 10.1038/s41586-025-08593-z

**Published:** 2025-03-05

**Authors:** Maximilian Dax, Stephen R. Green, Jonathan Gair, Nihar Gupte, Michael Pürrer, Vivien Raymond, Jonas Wildberger, Jakob H. Macke, Alessandra Buonanno, Bernhard Schölkopf

**Affiliations:** 1 Max Planck Institute for Intelligent Systems, Tübingen, Germany; 2 ETH Zurich, Zurich, Switzerland; 3 ELLIS Institute Tübingen, Tübingen, Germany; 4 School of Mathematical Sciences, University of Nottingham, Nottingham, UK; 5 Max Planck Institute for Gravitational Physics (Albert Einstein Institute), Potsdam, Germany; 6 Department of Physics, University of Maryland, College Park, MD USA; 7 Department of Physics, University of Rhode Island, Kingston, RI USA; 8 Center for Computational Research, University of Rhode Island, Kingston, RI USA; 9 Gravity Exploration Institute, Cardiff University, Cardiff, UK; 10 Machine Learning in Science, University of Tübingen & Tübingen AI Center, Tübingen, Germany

**Keywords:** General relativity and gravity, Stars, Transient astrophysical phenomena, Mathematics and computing, Nuclear astrophysics

## Abstract

Mergers of binary neutron stars emit signals in both the gravitational-wave (GW) and electromagnetic spectra. Famously, the 2017 multi-messenger observation of GW170817 (refs. ^1,2^) led to scientific discoveries across cosmology^3^, nuclear physics^4–6^ and gravity^7^. Central to these results were the sky localization and distance obtained from the GW data, which, in the case of GW170817, helped to identify the associated electromagnetic transient, AT 2017gfo (ref. ^8^), 11 h after the GW signal. Fast analysis of GW data is critical for directing time-sensitive electromagnetic observations. However, owing to challenges arising from the length and complexity of signals, it is often necessary to make approximations that sacrifice accuracy. Here we present a machine-learning framework that performs complete binary neutron star inference in just 1 s without making any such approximations. Our approach enhances multi-messenger observations by providing: (1) accurate localization even before the merger; (2) improved localization precision by around 30% compared to approximate low-latency methods; and (3) detailed information on luminosity distance, inclination and masses, which can be used to prioritize expensive telescope time. Additionally, the flexibility and reduced cost of our method open new opportunities for equation-of-state studies. Finally, we demonstrate that our method scales to long signals, up to an hour in length, thus serving as a blueprint for data analysis for next-generation ground- and space-based detectors.

## Abstract (web summary)

Analysis of gravitational waves from merging binary neutron stars was accelerated using machine learning, enabling full low-latency parameter estimation and enhancing the potential for multi-messenger observations.

## Main

The fast and accurate inference of binary neutron stars (BNSs) from gravitational-wave (GW) data is a critical challenge facing multi-messenger astronomy. For a BNS, the GW signal is visible by the Laser Interferometer GW Observatory (LIGO)–Virgo GW Interferometer (Virgo)–Kamioka GW Detector (KAGRA) (collectively, LVK)^9–11^ observatories minutes before any electromagnetic counterpart. The GW encodes information on the source characterization, distance, sky location and orientation necessary for pointing and prioritizing optical telescopes. However, the length of BNS signals makes conventional Bayesian inference techniques^12,13^ too slow to be useful in low-latency applications. Instead, once a GW signal is identified by detection pipelines^14,15^, approximate algorithms are used for providing initial alerts (for example, BAYESTAR^16^, which uses the signal-to-noise ratio (SNR) time series rather than the complete strain data and gives localization in seconds). Other methods focus on accelerating likelihood evaluations without incurring loss of precision (for example, using reduced-order quadratures), with the state-of-the-art delivering localization in 6 min and full inference in 2 h (ref. ^17^).

Simulation-based machine learning offers a powerful alternative for GW inference (see Methods for related work). With simulation-based inference (SBI)^18^, neural networks are trained to encode probabilistic estimates of astrophysical source parameters conditional on data. Trained networks then enable extremely fast analysis for new datasets, amortizing upfront training costs across observations. In past work, we developed the deep inference for GW observations (DINGO) framework for binary black holes (BBHs)^19,20^, which performs accurate inference in seconds, including strong accuracy guarantees when coupled with importance sampling. However, when applied to BNSs, machine-learning approaches, such as DINGO, are beset by the same challenges facing traditional methods because of long signal durations. Indeed, DINGO becomes unreliable even for low-mass BBHs (chirp masses ≲15 *M*_⊙_) with signals longer than roughly 16 s. A BNS lasts for hundreds of seconds for the LVK and will reach hours for next-generation detectors (for example, Cosmic Explorer^21^ and Einstein Telescope^22^). From the neural-network perspective, this corresponds to time or frequency series input with up to tens of millions of dimensions—a thousand-fold increase over BBH.

**Fig. 1:**
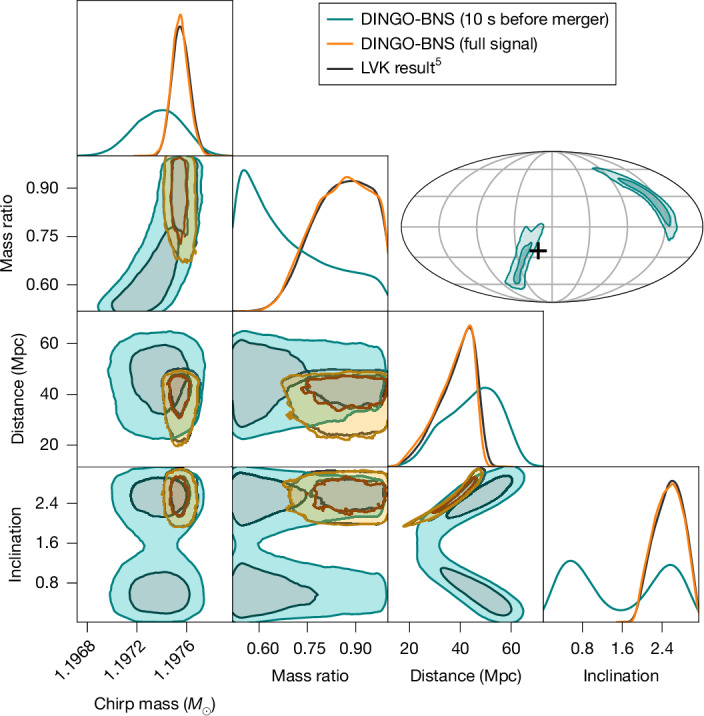
Real-time inference of GW170817. DINGO-BNS estimates all BNS parameters in just 1 s (orange, 10.8% sample efficiency), reproducing LVK results^5^ (black, 0.1% typical efficiency) three orders of magnitude faster than existing methods^17,33,39^. DINGO-BNS can also analyse partial data before the merger occurs (teal, 78.9% efficiency). Fast analysis results are crucial for directing electromagnetic searches for prompt, or even precursor, signals. Note that GW170817 overlapped with a loud glitch, which could explain why the true sky position lies in the tail of the pre-merger distribution.

**Fig. 2:**
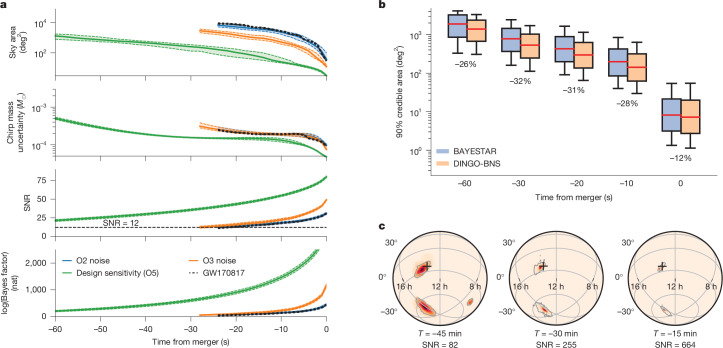
Pre-merger inference with DINGO-BNS. **a**, Evolution of pre-merger estimates for GW170817 (black) and GW170817-like simulations injected into different noise levels (colours). We display the 90% credible sky area, the standard deviation of the chirp mass, the accumulated signal-to-noise and the log(Bayes factor) in the natural unit of information (nat) comparing the signal and noise models. All of these quantities are inferred with a latency of around 1 s. Dotted lines represent the 10th/90th percentiles. We impose a minimum SNR^17^ of 12. **b**, Sky localization area at 90% credible level for various pre-merger times compared against BAYESTAR. The boxplots display the medians (percentage changes indicated), quartiles and 10th/90th percentiles. DINGO-BNS localization is consistently more precise. **c**, Pre-merger sky localization for a GW170817-like event injected into Cosmic Explorer noise, using a minimum frequency of 6 Hz. The black marker indicates the injection coordinates and the grey outline is the 90% credible area.

In this study, we overcome these challenges by leveraging perturbative BNS physics information to simplify and compress the data. However, this simplification requires approximate knowledge of the source itself and is hence valid only over a small portion of the parameter space. We solve this problem using a new algorithm, called prior conditioning, which enables us to construct networks that can be adapted at inference time to subsets of the prior volume. Our new framework, called DINGO-BNS, makes no (practically relevant) approximation and takes just 1 s for accurate inference of all 17 BNS parameters (Fig. 1). Using DINGO-BNS, we can also infer all of these parameters minutes before the merger based on partial inspiral-only information—estimates that can be continuously updated as more data become available (Fig. 2a). Near-real-time or pre-merger alerts can then be provided to astronomers, facilitating potential discoveries of precursor and prompt electromagnetic counterparts^23–25^.

Our results are faster and more complete than any existing low-latency algorithm, with the accuracy of offline parameter estimation codes. Compared to BAYESTAR, we achieve median reductions in the size of the 90% credible sky region of 30% (Fig. 2b). Finally, DINGO-BNS exhibits excellent scaling to longer signals (Methods), and we demonstrate next-generation detector pre-merger inference for signals up to an hour in length (Fig. 2c).

## DINGO-BNS

For given GW data, **d**, we characterize the source in terms of the posterior probability distribution, *p*(**θ**|**d**), over BNS parameters, **θ**. Parameters include component masses (two), spins (six), orientation, sky position (two), luminosity distance, polarization, time and phase of coalescence and (in contrast to black holes) tidal deformabilities (two). Following our past work^19^, we use simulated GW datasets to train a density estimation neural network, *q*(**θ**|**d**) (a normalizing flow), to approximate *p*(**θ**|**d**). Once trained, the inference for new **d** simply requires sampling **θ** ~ *q*(**θ**|**d**). We obtain asymptotically exact results by augmenting samples with importance weights using the GW likelihood function^26^. This framework, called DINGO-IS^20^, has been successfully applied to black hole mergers. However, the length of BNS signals renders the naïve transfer of machine-learning methods impossible.

**Fig. 3:**
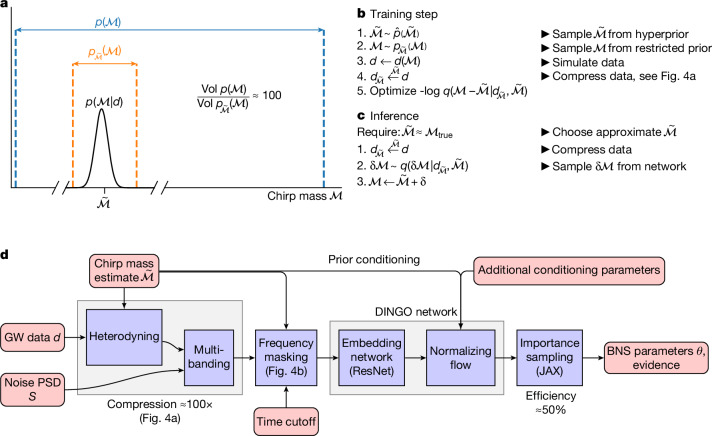
Prior conditioning and method flowchart. **a**, For a typical event, the chirp mass posterior (black) is tightly constrained compared to the prior (blue), so a tighter prior (orange) that still covers the posterior is sufficient for inference. In addition, the narrow prior can simplify the analysis. Our prior-conditioning technique therefore trains a single neural network that can be tuned at inference time to an event-specific prior. **b**, Training is accomplished by simulating data from randomly chosen narrow priors, ${p}_{\widetilde{{\mathcal{M}}}}({\mathcal{M}})$, each parametrized by a reference chirp mass, $\widetilde{{\mathcal{M}}}$, on which the network is also conditioned. Prior conditioning enables prior-specific heterodyning based on $\widetilde{{\mathcal{M}}}$, followed by multibanding compression (Fig. 4a), effectively simplifying the data distribution that the model must learn and reducing its dimensionality. **c**, At inference time, an initial chirp mass estimate, $\widetilde{{\mathcal{M}}}$, determines the event-specific prior and compression. **d**, Prior conditioning and the other technical innovations are integrated into a single neural network that can be trained end-to-end and produce 10^5^ weighted samples per second, with typical sampling efficiencies of 50%.

To tackle this challenge, DINGO-BNS makes several innovations (Fig. 3d), including using knowledge of specific BNS signal morphology to compress data in a non-lossy way, conditioning the network on the compressor using prior conditioning, frequency masking based on the pre-merger time and chirp mass, and conditioning on parameter subsets for incorporating multi-messenger information or expectations from nuclear models. The philosophy underlying our approach is that the full BNS problem is too hard for existing neural architectures, so we divide the parameter and data spaces into manageable portions based on known physical information. We then combine all of these variable design choices into a single network. By passing relevant control parameters at inference time, the network can be tuned to the context at hand.

## Data compression and prior conditioning

**Fig. 4:**
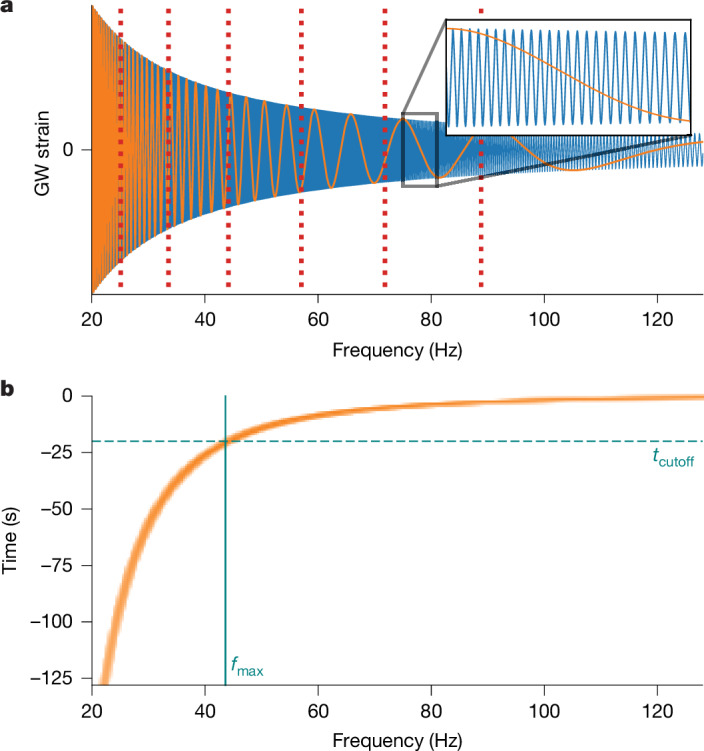
Compression and frequency masking. **a**, We compress data by a factor of around 100 by first factoring out (that is, heterodyning) the predominant phase evolution of the signal (blue). The resulting simplified signal (orange) is down-sampled in resolution, reducing data dimensionality (coarser resolution at high frequencies; bands indicated by dotted red lines). **b**, To enable pre-merger inference, we mask out the strain frequency series according to the cutoff time.

We adapted two GW analysis techniques to the SBI context—heterodyning^27^ to simplify the data and multibanding^28,29^ to reduce the data dimension without loss of information. During the long inspiral period, a BNS signal exhibits a ‘chirp’, with phase evolution (to leading order in the post-Newtonian expansion^30^),

1 $${\varphi }(f;{\mathcal{M}})=\frac{3}{128}{\left(\frac{{\rm{\pi }}G{\mathcal{M}}f}{{c}^{3}}\right)}^{-5/3},$$

where *f* is the frequency, *c* is the speed of light in vacuum and ${\mathcal{M}}$ = (*m*_1_*m*_2_)^3*/*5^*/*(*m*_1_ + *m*_2_)^1*/*5^ is the chirp mass of the system, with *m*_1_ and *m*_2_ being the component masses. Given an approximation, $\widetilde{{\mathcal{M}}}$, to the chirp mass, we heterodyne the (frequency-domain) data by multiplying by ${{\rm{e}}}^{{\rm{i}}{\boldsymbol{\varphi }}({\boldsymbol{f}};\widetilde{{\mathcal{M}}})}$, reducing the number of oscillations in the signal by several orders of magnitude (Fig. 4a). Given the heterodyned data, we apply multibanding by partitioning the domain into (empirically determined) frequency bands and coarsening the resolution in higher bands, such that the (heterodyned) signal is preserved.

Because the compression described requires $\widetilde{{\mathcal{M}}}$ to approximate the chirp mass, it cannot be done across the entire BNS prior volume using a single $\widetilde{{\mathcal{M}}}$ value. Therefore, DINGO-BNS uses prior conditioning to restrict to an event-specific prior over which data are compressed. The restricted volume additionally simplifies the density estimation task. By conditioning on the choice of restriction, prior conditioning trains a network that is tunable to this choice, but otherwise applicable over the whole volume (Fig. 3a). Inference requires an estimate, $\widetilde{{\mathcal{M}}}$, of the chirp mass, ${\mathcal{M}}$, which can be determined quickly by sweeping across the prior (Methods).

## Frequency masking

In contrast to past work, DINGO-BNS also allows strain frequency series with varying minimum and maximum frequencies (*f*_min_ and *f*_max_, respectively). For a given analysis, *f*_min_ is chosen based on $\widetilde{{\mathcal{M}}}$ and the segment duration as the minimum frequency present in the signal in a given GW-detector network. This masking is necessary for consistency with frequency-domain waveform models, which assume infinite duration. Choosing *f*_max_, by contrast, determines the end time of the data stream analysed to enable pre-merger inference (Fig. 4b and Methods).

## Conditioning on parameter subsets

The DINGO-BNS framework (and SBI in general) allows considerable flexibility in terms of quickly marginalizing over, and conditioning on, parameters. Conditioning on a parameter allows us to set it to a fixed value—for example, to incorporate knowledge of that parameter from other sources. In our study, we trained DINGO-BNS networks conditioned on the sky position—that is, we learned *p*(**θ**\{*α*,*δ*}|**d**,*α*,*δ*), where *α* and *δ* denote the right ascension and declination, respectively. Such a network allows us to incorporate precise multi-messenger localization to obtain tighter constraints on the remaining parameters, potentially enabling real-time feedback on whether optical candidates should be prioritized for detailed spectroscopy^31^. In this way, DINGO-BNS can enable new modes of interaction between GW and electromagnetic observers, potentially transforming how we prioritize and respond to multi-messenger events. We have also explored parameter-conditioning to accelerate offline nuclear equation-of-state (EOS) analyses (Methods).

## Experiments

We generated training data using simulated BNS waveforms (including spin-precession and tidal contributions, but without higher angular multipoles^32^) with additive stationary Gaussian detector noise. When relevant, networks are also trained with power spectral density (PSD)-conditioning to enable instant tuning to noise levels at the time of an event. At inference time, we validate and correct results using importance sampling, thus guaranteeing their accuracy, provided a sufficient effective sample size is obtained^20^. We accelerate the importance sampling step using JAX waveform and likelihood implementations^33–35^.

We performed four studies using DINGO-BNS: (1) a pre-merger analysis of the first BNS detected—GW170817—as well as equivalent injections (simulated datasets) at varying noise levels; (2) a pre-merger analysis of a range of injections in LVK design sensitivity noise; (3) an after-merger analysis of the two detected BNS events—GW170817 and GW190425—reproducing published LVK results; and (4) a pre-merger analysis of injections in Cosmic Explorer noise (with a minimum frequency of 6 Hz, corresponding to an hour-long signal). We use the importance sampling efficiency as a primary performance metric, finding average values of 63.3%, 47.0%, 31.0% and 35.6% in experiments (1), (2), (3) and (4), respectively. With these high efficiencies, inference for 10^4^ effective samples takes roughly 1 s on an H100 GPU (Methods). Efficiencies are generally higher for pre-merger, probably because the waveform morphology is simplest in the early inspiral.

## Discussion

Prior conditioning works well for BNS inference, and it could be extended to address further challenges in GW astronomy (for example, the isolation of events from overlapping backgound signals in next-generation detectors) and other scientific domains. In the future, we would like to explore our prior-conditioning approach to data compression for black hole–neutron star systems and low-mass BBHs. This is non-trivial because such systems can emit GWs in higher angular radiation multipoles (that is, beyond the (*l*,*m*) = (2,2) mode that we assume here), which evolve according to integer multiples of (1), and so would require an improved heterodyning algorithm to factor out the chirp. Higher modes are not present in BNS signals, because the stars are very nearly equal in mass.

Another exciting prospect for SBI is a more realistic treatment of detector noise. Indeed, because BNS inspirals have long durations, noise non-stationarities and non-Gaussianities are more likely to manifest. Currently, DINGO-BNS assumes stationary Gaussian noise and is supplied with an estimate of the PSD (possibly resulting in additional latency for data preparation, see Methods). However, by training on realistic detector noise, our approach can, in principle, learn to fully characterize the noise jointly with the signal, including any deviations from stationarity and Gaussianity. This approach is akin to on-source PSD and glitch modelling^36^, but allows more general noise and automatically marginalizes over uncertainties. Initial steps in this direction have already been taken for intermediate-mass BBHs^37^. Improved noise treatments, such as those afforded by SBI, will become crucial for reducing systematic error as detectors become more sensitive^38^.

Finally, although DINGO-BNS is intended to be used for parameter estimation following a trigger by dedicated search pipelines, its speed opens up the possibility of being run continuously on all data as they are taken. Either the SNR or Bayesian evidence time series generated by DINGO-BNS could then be used as a detection statistic, forming an end-to-end detection and parameter estimation pipeline. To implement this would require calibrating these statistics to determine false alarm rates, as well as careful comparisons against existing algorithms to establish efficacy.

## Methods

### Machine-learning framework

The Bayesian posterior, *p*(**θ**|**d**) = *p*(**d**|**θ**)*p*(**θ**)*/p*(**d**), is defined in terms of a prior *p*(**θ**) and a likelihood *p*(**d**|**θ**). For GW inference, the likelihood is constructed by combining models for waveforms and detector noise. The Bayesian evidence, *p*(**d**), corresponds to the normalization of the posterior and can be used for model comparison.

Our framework is based on neural posterior estimation (NPE)^40–42^, which trains a density estimation neural network, *q*(**θ**|**d**), to estimate *p*(**θ**|**d**). We parameterize *q*(**θ**|**d**) with a conditional normalizing flow^43,44^. Training minimizes the loss *L* = E*p*_(**θ**,**d**)_[−log *q*(**θ**|**d**)], where the expectation value is computed across a dataset (**θ**_*i*_,**d**_*i*_) of parameters, **θ**_*i*_ ~ *p*(**θ**), paired with corresponding likelihood simulations, **d**_*i*_ ~ *p*(**d**|**θ**_*i*_). After training, *q*(**θ**|**d**) serves as a surrogate for *p*(**θ**|**d**) and inference for any observed data, **d**_o_, can be performed by sampling **θ** ~ *q*(**θ***|***d**_o_). With DINGO^19,20^ using a group-equivariant formulation of NPE (GNPE^19,45^), the GW data are simplified by aligning coalescence times in the different detectors. However, this comes at the cost of longer inference times, so we do not use GNPE for DINGO-BNS.

At inference, we correct for potential inaccuracies of *q*(**θ**|**d**) using importance sampling^20^, by assigning weight, *w*_*i*_ = *p*(**d**|**θ**_*i*_)*p*(**θ**_*i*_)*/q*(**θ**_*i*_|**d**), to each sample, **θ**_*i*_ ~ *q*(**θ**_*i*_|**d**). A set of *n* weighted samples (*w*_*i*_,**θ**_*i*_) corresponds to ${n}_{\text{eff}}={\left({\sum }_{i}{w}_{i}\right)}^{2}\,/\,\left({\sum }_{i}{w}_{i}^{2}\right)$ effective samples from the posterior, *p*(**θ**|**d**). This reweighting enables asymptotically exact results and the sample efficiency, *ϵ* = *n*_eff_*/n*, serves as a performance metric. The normalization of the weights further provides an unbiased estimate of the Bayesian evidence, $p({\bf{d}})=({\sum }_{i}{w}_{i})/n$.

Below, we describe in more detail the technical innovations of DINGO-BNS that enable scaling of this framework to BNS signals.

#### Prior conditioning

**Extended Data Fig. 1:**
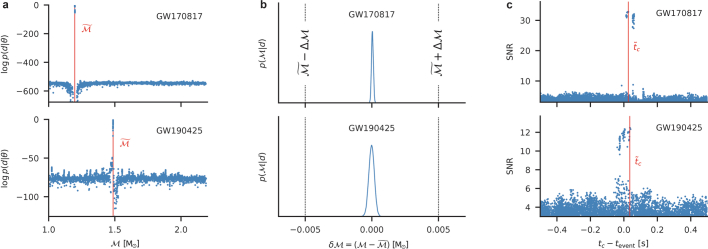
DINGO-BNS scan estimates the chirp mass and merger time. (a) Log likelihoods generated from a scan over different values of $\widetilde{{\mathcal{M}}}$ with a DINGO-BNS network. The final $\widetilde{{\mathcal{M}}}$ is chosen as the maximum likelihood ${\mathcal{M}}$ (red line; $\widetilde{{\mathcal{M}}}$ = 1.1975 M_⊙_ for GW170817, $\widetilde{{\mathcal{M}}}$ = 1.4868 M_⊙_ for GW190425). (b) Posterior marginal *p*(${\mathcal{M}}$*|***d**). The prior (dashed lines) determined by the scan from (a) fully covers the marginal. (c) A combined scan over ${\mathcal{M}}$ and *t*_c_ successfully identifies GW170817 (with ${\hat{t}}_{{\rm{c}}}$ = 1187008882.43) and GW190425 (with ${\hat{t}}_{{\rm{c}}}$ = 1240215503.04).

An NPE model, *q*(**θ**|**d**), estimates the posterior, *p*(**θ**|**d**), for a fixed prior, *p*(**θ**). Choosing a broad prior enhances the general applicability of the NPE model, but it also implies worse tuning to specific events (for which smaller priors may be sufficient). This is a general trade-off in NPE, but it is particularly notable for BNS inference, where typical events constrain the chirp mass to around 10^*−*3^ of the prior volume. Thus, for an individual BNS event, a tight chirp mass prior would have been sufficient (Extended Data Fig. 1b) and moreover would have enabled effective heterodyning^27,46,47^. However, to cover generic BNS events, we need to train the NPE network with a large prior (Extended Data Table 2).

We resolve this trade-off with a new technique called prior conditioning. The key idea is to train an NPE model with multiple different (restricted) priors simultaneously. Training a prior-conditioned model requires hierarchical sampling:

2 $${\boldsymbol{\theta }} \sim {{p}}_{{\boldsymbol{\rho }}}({\boldsymbol{\theta }}),{\boldsymbol{\rho }} \sim \widehat{p}({\boldsymbol{\rho }}),$$

where *p*_**ρ**_(**θ**) is a prior family parameterized by **ρ** and $\widehat{p}({\boldsymbol{\rho }})$ is a corresponding hyperprior. We additionally condition the NPE model, *q*(**θ**|**d**,**ρ**), on **ρ**. This model can then perform inference for any desired prior, *p*_**ρ**_(**θ**), by simply providing the corresponding **ρ**. This effectively amortizes the training cost over different choices of the prior. On each of the restricted priors, we are furthermore allowed to transform the data in a **ρ**-dependent way. This is because, having been conditioned on the prior choice, the network has all the information necessary to properly interpret the transformed data. We use this freedom to heterodyne the GW strain with respect to the approximate chirp mass.

To apply prior conditioning for the chirp mass, ${\mathcal{M}}$, we use a set of priors, ${p}_{\widetilde{{\mathcal{M}}}}({\mathcal{M}})={U}_{{m}_{1},{m}_{2}}(\widetilde{{\mathcal{M}}}-\Delta {\mathcal{M}},\widetilde{{\mathcal{M}}}+\Delta {\mathcal{M}})$. Here, *U*_*m*1,*m*2_(${\mathcal{M}}$_min_, ${\mathcal{M}}$_max_) denotes a distribution over ${\mathcal{M}}$ with support, [${\mathcal{M}}$_min_, ${\mathcal{M}}$_max_], within which component masses, *m*_1_, *m*_2_, are uniformly distributed. We use a fixed ∆${\mathcal{M}}$ = 0.005 *M*_⊙_ and choose a hyperprior, $\hat{p}(\widetilde{{\mathcal{M}}})$, covering the expected range of ${\mathcal{M}}$ for LVK detections of BNS (Extended Data Table 2). Because ∆${\mathcal{M}}$ is small, $\widetilde{{\mathcal{M}}}$ is a good approximation for any ${\mathcal{M}}$ within the restricted prior, ${p}_{\widetilde{{\mathcal{M}}}}({\mathcal{M}})$, and we can thus use $\widetilde{{\mathcal{M}}}$ for heterodyning. The resulting model, $q({\boldsymbol{\theta }}| {{\bf{d}}}_{\widetilde{{\mathcal{M}}}},\widetilde{{\mathcal{M}}})$, can then perform inference with event-optimized heterodyning and priors (via the choice of appropriate $\widetilde{{\mathcal{M}}}$), but is nevertheless applicable to the entire range of the hyperprior.

Inference results are independent of $\widetilde{{\mathcal{M}}}$ as long as the posterior, *p*(${\mathcal{M}}$*|***d**), is fully covered by [$\widetilde{{\mathcal{M}}}$ − ∆${\mathcal{M}}$, $\widetilde{{\mathcal{M}}}$ + ∆${\mathcal{M}}$]. For BNS, *p*(${\mathcal{M}}$*|***d**) is typically tightly constrained and we can use a coarse estimate of ${\mathcal{M}}$ for $\widetilde{{\mathcal{M}}}$. This can either be taken from a GW search pipeline or rapidly computed from $q({\boldsymbol{\theta }}| {{\bf{d}}}_{\widetilde{{\mathcal{M}}}},\widetilde{{\mathcal{M}}})$ itself by sweeping the hyperprior (see below). Note that, for shorter GW signals from black hole mergers, *p*(${\mathcal{M}}$*|***d**) is generally less well constrained. The transfer of prior conditioning would thus require larger (and potentially flexible) values of ∆${\mathcal{M}}$. Alternatively, the prior range can be extended at inference time by iterative Gibbs sampling of ${\mathcal{M}}$ and $\widetilde{{\mathcal{M}}}$, similar to the GNPE algorithm^19,45^.

Prior conditioning is a general SBI technique that enables a choice of prior at inference time. This can also be achieved with sequential NPE^40–42,48^. However, in contrast to prior conditioning, these techniques require simulations and retraining for each observation, resulting in more expensive and slower inference. We here use prior conditioning with priors of fixed width for the chirp mass, and optional additional conditioning on fixed values for other parameters (corresponding to Dirac delta priors). Extension to more complicated priors and hyperpriors is straightforward.

#### Independent estimation of chirp mass and merger times

Running DINGO-BNS requires an initial estimate of the chirp mass, ${\mathcal{M}}$ (to determine $\widetilde{{\mathcal{M}}}$ for the network), and the merger time, *t*_c_ (to trigger the analysis). Matched filter searches can identify the presence of a compact binary signal and its chirp mass and merger time in low latency^14,15,49–51^. Specialized early warning searches are designed to produce output before the coalescence can further provide a rough indication of sky position and distance^52–54^. When available, the output of such pipelines can be used to trigger a DINGO analysis and provide estimates for ${\mathcal{M}}$ and *t*_c_.

We here describe an alternative independent approach of obtaining these parameters, using only the trained DINGO-BNS model. We compute $\widetilde{{\mathcal{M}}}$ by sweeping the entire hyperprior, $\hat{p}(\widetilde{{\mathcal{M}}})={U}_{{m}_{1},{m}_{2}}({\widetilde{{\mathcal{M}}}}_{\min },{\widetilde{{\mathcal{M}}}}_{\max })$. Specifically, we run DINGO-BNS with a set of prior centres,

3 $${\mathop{{\mathcal{M}}}\limits^{ \sim }}_{i}={\mathop{{\mathcal{M}}}\limits^{ \sim }}_{min}+i\Delta {\mathcal{M}},$$

where *i* takes integer values between 0 and ($\widetilde{{\mathcal{M}}}$_max_ − $\widetilde{{\mathcal{M}}}$_min_)*/*∆${\mathcal{M}}$. The inference models in this study were trained with hyperprior ranges of up to [1.0,2.2] *M*_⊙_. For ∆${\mathcal{M}}$ = 0.005 *M*_⊙_, we can thus cover the entire global chirp mass range using 241 (overlapping) local priors. We run DINGO-BNS for all local priors, ${\widetilde{{\mathcal{M}}}}_{i}$, in parallel, with 10 samples per ${\widetilde{{\mathcal{M}}}}_{i}$. This requires a DINGO-BNS inference of only a few thousand samples, which takes less than 1 s. We use the chirp mass, ${\mathcal{M}}$, of the maximum likelihood sample as the prior centre, $\widetilde{{\mathcal{M}}}$, for the analysis (Extended Data Fig. 1a). Note that the exact choice of $\widetilde{{\mathcal{M}}}$ does not matter, as long as the inferred posterior is fully covered by [$\widetilde{{\mathcal{M}}}$ − ∆${\mathcal{M}}$, $\widetilde{{\mathcal{M}}}$ + ∆${\mathcal{M}}$] (Extended Data Fig. 1b).

The merger time, *t*_c_, can be inferred by continuously running this $\widetilde{{\mathcal{M}}}$ scan on the input data stream, sliding the *t*_c_ prior in real time over the incoming data. With inference times of 1 s, continuous analysis can be achieved on just a few parallel computational nodes (or even a single node when also parallelizing over the *t*_c_ grid), constantly running on the input data stream. Event candidates can then be identified by analysing the SNR, triggering upon exceeding some defined threshold (Extended Data Fig. 1c). This scan can be performed at an arbitrary (but fixed) time before the merger.

This scan successfully estimates ${\mathcal{M}}$ and *t*_c_ for both real BNS events (Extended Data Fig. 1). However, we have not tested this at a large scale on detector noise to compute false alarm rates because DINGO-BNS is primarily intended for parameter estimation. Existing search and early warning pipelines are probably more robust for event identification, particularly in the presence of non-stationary detector noise.

#### Frequency multibanding

**Extended Data Fig. 2:**
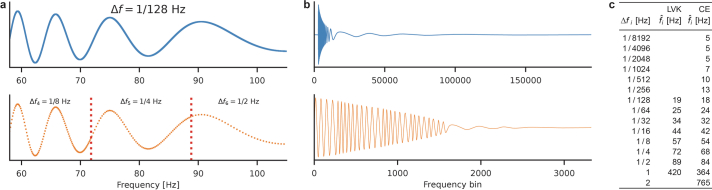
Frequency multibanding. (a) The period of (heterodyned) GW signals decreases with increasing frequency. The native frequency resolution (blue) thus oversamples the signal at high frequencies. Frequency multibanding (band boundaries indicated by dotted red lines) adapts to the signal variation, decreasing the resolution at higher frequencies (orange). (b) The multibanded domain therefore requires fewer frequency bins, and the signal variation is more homogeneous across bins. (c) Multibanded frequency domain partitions for LVK (*f*_min_ = 19.4 Hz, compression factor ≈60) and CE (*f*_min_ = 5 Hz, compression factor ≈650) experiments. We use a smaller chirp mass prior for the CE experiments (Extended Data Table 2), which allows a slightly coarser resolution compared to LVK (corresponding to lower ${\hat{f}}_{i}$). The first two bands for CE are skipped entirely, which is a consequence of the reduced signal variation with heterodyning.

Although the native resolution of a frequency series is determined by the duration, *T*, of the corresponding time series, (∆*f* = 1*/T*), we can average adjacent frequency bins wherever the signal is roughly constant. This enables data compression with only negligible loss of information. Here we use frequency multibanding, which divides the frequency range, [*f*_min_, *f*_max_], into *N* bands of decreasing resolution. Frequency band *i* covers the range $[\,{\hat{f}}_{i}\,,\,{\hat{f}}_{i+1}\,]$ with ∆*f*_*i*_ = 2^*i*^∆*f*_0_, where ${\hat{f}}_{0}\,=\,{f}_{\min }$, ${\hat{f}}_{N}\,=\,{f}_{\max }$ and ∆*f*_0_ is the native resolution of the frequency series. Within band *i*, the multibanded domain thus compresses the data by a factor of 2^*i*^ (Extended Data Fig. 2), which is achieved by averaging 2^*i*^ sequential bins from the original frequency series (called decimation). To achieve optimal compression, we empirically choose the smallest possible nodes, ${\hat{f}}_{i}$, for which GW signals are still fully resolved. Specifically, we simulate a set of 10^3^ heterodyned GW signals and demand that every period of these signals is covered by at least 32 bins in the resulting multibanded frequency domain. This is done before generating the training dataset, and the multibanded domain then remains fixed during dataset generation and training. The optimized resolution achieves compression factors between 60 and 650 (Extended Data Fig. 2c).

Traditionally, multibanding has been used without additional heterodyning—an approach we could also apply to DINGO-BNS. However, this would lead to lower compression factors, ranging from 14 to 56. More importantly, using multibanding alone would result in substantially more complicated input data to the DINGO-BNS network. Indeed, heterodyning enables additional truncation of the waveform singular value decomposition used in initializing the first network layer^19^. For LVK data, this results in a reduction from roughly 1,800 to 200 basis elements, thereby simplifying the learning task.

Care needs to be taken that the approximations are valid in the presence of detector noise. We now investigate how multibanding affects data simulation (for training) and the likelihood (for importance sampling).

##### Data simulation

The GW data are simulated as the sum of a signal and detector noise, **d** = **h**(**θ**) + **n**. The detector noise in frequency bin *j* is given by

4 $${n}_{j}\sim {\mathcal{N}}(0,{\sigma }\sqrt{{S}_{j}}),{\sigma }=\sqrt{\frac{w}{4\Delta f}},$$

where **S** denotes the detector noise PSD, and *σ* takes into account the frequency resolution and the Tukey window factor, *w*. Note that **n** is a complex frequency series, which we ignore in our notation, as the considerations here hold for real and imaginary parts individually. It is conventional to work with whitened data,

5 $${d}_{j}^{\text{w}}={h}_{j}^{\text{w}}({\boldsymbol{\theta }})+{n}_{j}^{\text{w}}=\frac{{h}_{j}({\boldsymbol{\theta }})+{n}_{j}}{\sqrt{{S}_{j}}},$$

in which case ${n}_{j}^{\text{w}}\sim {\mathcal{N}}(0,{\sigma })$.

We convert to the multibanded frequency domain by averaging sets of *N*_*i*_ = 2^*i*^ bins,

6 $$\bar{{d}_{j}^{\text{w}}}=\frac{1}{{N}_{i}}\mathop{\sum }\limits_{k={m}_{j}}^{{m}_{j}+{N}_{i}-1}({h}_{k}^{\text{w}}+{n}_{k}^{\text{w}})=\bar{{h}_{j}^{\text{w}}}+\bar{{n}_{j}^{\text{w}}},$$

where *j* denotes the bin in the multibanded domain, *m*_*j*_ denotes the starting index of the decimation window for *j* in the native domain and *i* indexes the frequency band associated with *j*. Because $\bar{{n}_{j}^{\text{w}}}$ is an average of *N*_*i*_ Gaussian random variables with standard deviation *σ*, it follows that ${n}_{j}^{\text{w}}$ is also Gaussian with standard deviation,

7 $${{\sigma }}_{i}={\sigma }\,/\,\sqrt{{N}_{i}}=\sqrt{\frac{w}{4\Delta f{N}_{i}}}=\sqrt{\frac{w}{4\Delta {f}_{i}}}.$$

We can thus simulate the detector noise directly in the multibanded domain by updating *σ* → *σ*_*i*_, corresponding to ∆*f* → ∆*f*_*i*_. For the whitened signal we find

8 $$\overline{{h}_{j}^{\text{w}}}=\frac{1}{{N}_{i}}\mathop{\sum }\limits_{k={m}_{j}}^{{m}_{j}+{N}_{i}-1}\frac{{h}_{k}}{\sqrt{{S}_{k}}}\approx \bar{{h}_{j}}\mathop{\sum }\limits_{k={m}_{j}}^{{m}_{j}+{N}_{i}-1}\frac{1}{\sqrt{{S}_{k}}},$$

assuming an approximately constant signal, **h**, within the decimation window, $\bar{{h}_{j}}$ *≈ h*_*k*_,∀*k* ∈ [*m*_*j*_,*m*_*j*_ + *N*_*i*_ − 1]. For frequency-domain waveform models, we can thus directly compute the signal $\bar{{h}_{j}}$ in the multibanded domain by simply evaluating the model at frequencies $\bar{{f}_{j}}$.

In summary, we can directly generate BNS data in the multibanded frequency domain by: (1) updating the noise standard deviation according to the multibanded resolution; (2) appropriately decimating noise PSDs; and (3) computating signals and noise realizations in the compressed domain. These operations are carefully designed to be consistent with the data processing of real BNS observations, which for DINGO-BNS are first whitened in the native domain and then decimated to the multibanded domain. This process relies on the assumption that signals are constant within decimation windows, and we ensure that this is (approximately) fulfilled when determining the multibanded resolution. Indeed, for signals generated directly in the multibanded domain, we find mismatches of at most around 10^*−*7^ when comparing to signals that are properly decimated from the native domain.

##### Likelihood evaluations

We also use frequency multibanding to evaluate the likelihood for importance sampling. The standard Whittle likelihood used in GW astronomy^26^ reads:

9 $$\log p({\bf{d}}| {\boldsymbol{\theta }})=-\frac{1}{2}\sum _{k}\frac{{| {d}_{k}^{\text{w}}-{h}_{k}^{\text{w}}({\boldsymbol{\theta }})| }^{2}}{{\sigma }^{2}},$$

up to a normalization constant. The sum extends over all bins, *k*, in the native frequency domain. Assuming a constant signal (as above) and PSD within each decimation window, we can directly compute the likelihood in the multibanded domain,

10 $$\log \,p({\bf{d}}| {\boldsymbol{\theta }})\approx -\frac{1}{2}\sum _{j}\frac{{| \bar{{d}_{j}^{\text{w}}}-\bar{{h}_{j}^{\text{w}}}({\boldsymbol{\theta }})| }^{2}}{{\sigma }_{i(j)}^{2}}.$$

The assumptions are not exactly fulfilled in practice—for additional corrections, see ref. ^29^. For importance sampling, we can always evaluate the exact likelihood in the native frequency domain instead. In this case, the result is no longer subject to any approximations, even if the DINGO-BNS proposal is generated with a network using multibanded data. With the full likelihood for GW170817, we found a sample efficiency of 11.0% with an inference time of 13 s for 50,000 samples. The deviation from the result obtained with the multibanded likelihood is negligible (Jensen–Shannon divergence of less than 5 *×* 10^*−*4^  nat for all parameters). This demonstrates that use of the multibanded resolution has no practically relevant impact on the results.

#### Frequency masking

**Extended Data Fig. 3:**
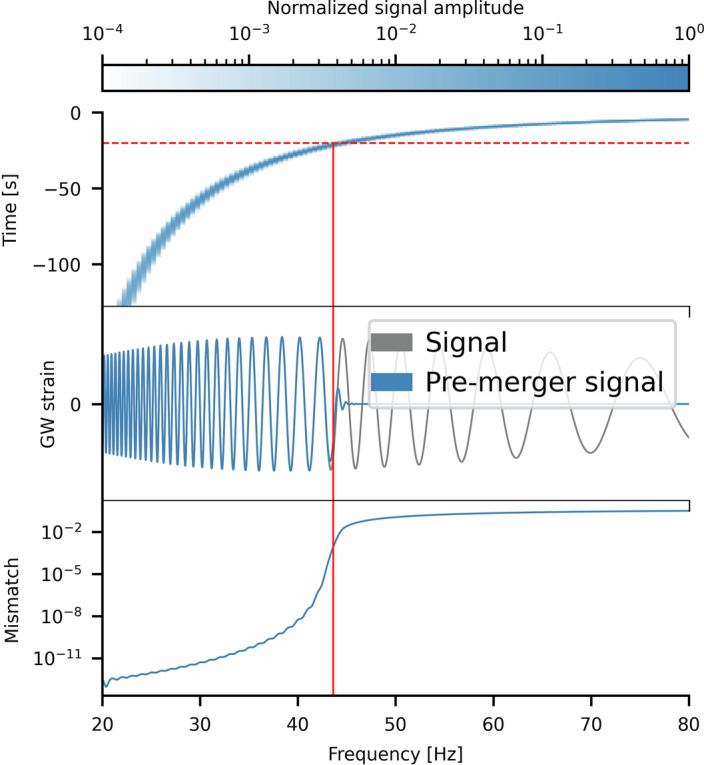
Time-domain truncation of BNS signals. Truncation at time *t*_max_ (red dashed line) before the merger can be approximated by truncation at a corresponding maximum frequency (red solid line) in frequency domain. Below frequency *f*_max_(*t*, ${\mathcal{M}}$), the truncated signal (blue in center panel) matches the original signal (gray). Above *f*_max_(*t*, ${\mathcal{M}}$), the amplitude of the truncated signal quickly approaches zero. We determine *f*_max_(*t*, ${\mathcal{M}}$) empirically, by allowing mismatches between truncated and original signals of at most 10^*−*3^ (lower panel). Analogously, truncation for *t < t*_min_ can be achieved by imposing a minimum frequency cutoff *f*_min_(*t*, ${\mathcal{M}}$).

Because the GW likelihood (and our framework) uses the frequency domain, but data are taken in the time domain, it is necessary to convert data by windowing and Fourier transforming. However, frequency domain waveform models assume infinite time duration, leading to inconsistencies with finite time segments, [*t*_min_, *t*_max_]. Because the frequency evolution of the inspiral is tightly constrained by the chirp mass, ${\mathcal{M}}$, we can compute boundaries, *f*_min_(*t*_min_, ${\mathcal{M}}$) and *f*_max_(*t*_max_, ${\mathcal{M}}$), such that the signals are not corrupted by the finite-duration effects within [*f*_min_, *f*_max_] and are negligibly small outside of that range (Extended Data Fig. 3).

We approximate the lower bound, *f*_min_(*t*_min_, ${\mathcal{M}}$), using the leading order in the post-Newtonian relationship between time and frequency,

11 $${f}_{0\text{PN}}(t,{\mathcal{M}})=\frac{1}{8{\rm{\pi }}}{\left(\frac{-t}{5}\right)}^{-3/8}{\left(\frac{G{\mathcal{M}}}{{c}^{3}}\right)}^{-5/8}.$$

For a network designed for fixed data duration, *T*, we set *f*_min_(*T*, ${\mathcal{M}}$) = *f*_0PN_(*−T*, ${\mathcal{M}}$) + *f*_buffer_ (we use *f*_buffer_ = 1 Hz for LVK and *f*_buffer_ = 0.5 Hz for next-generation detector setups).

For the upper bound, we found that *f*_0PN_(*t*, ${\mathcal{M}}$) is not sufficiently accurate. Instead, we determined *f*_max_(*t*, ${\mathcal{M}}$) empirically by simulating a set of signals (with parameters **θ** ~ *p*(**θ**)) and computing mismatches between signals with and without truncation at *t* > *t*_max_. For a given set of simulations, we choose *f*_max_(*t*, ${\mathcal{M}}$) as the highest frequency at which all mismatches are at the most 10^*−*3^. To avoid additional computation at inference time, we cache the results in a lookup table for *f*_max_(*t*, ${\mathcal{M}}$). The lookup table used here was generated with 20 waveforms per element. We verified for random elements that this matches the result obtained using 1,000 waveforms with an accuracy of approximately 0.1 Hz. For production use, the lookup table may need to be generated with more waveforms.

Both bounds depend on the chirp mass, ${\mathcal{M}}$, with the upper bound additionally depending on the pre-merger time. To enable inference for arbitrary configurations, we trained a single network with variable frequency bounds. During training, we computed *f*_min_(*T*, $\widetilde{{\mathcal{M}}}$) with the centre $\widetilde{{\mathcal{M}}}$ of the local chirp mass prior. The upper frequency bound, *f*_max_, is sampled randomly (uniform in frequency bins of the multibanded frequency domain) to allow arbitrary pre-merger times. Data outside of [*f*_min_, *f*_max_] are zero-masked.

Such masked networks can perform pre-merger inference. Given an alert (for example, from a detection pipeline) predicting a merger at time ${\widetilde{t}}_{{\rm{c}}}$ (in the future) with chirp mass $\widetilde{{\mathcal{M}}}$, DINGO-BNS uses the frequency range [*f*_min_(*T*, $\widetilde{{\mathcal{M}}}$), *f*_max_(*−*${\widetilde{t}}_{{\rm{c}}}$, $\widetilde{{\mathcal{M}}}$)]. The frequency domain data are further shifted by ${\widetilde{t}}_{{\rm{c}}}$, such that the prior *p*(*t*_c_) is centred around ${\widetilde{t}}_{{\rm{c}}}$. The resulting pre-merger posterior can then be used to update the alert predictions ($\widetilde{{\mathcal{M}}}$, ${\widetilde{t}}_{{\rm{c}}}$) (for example, as the centre of the inferred posterior marginals) and to trigger a new DINGO-BNS analysis with the new strain data that became available in the meantime. Such iterative posterior updates allow continuous optimization of the prior centres ($\widetilde{{\mathcal{M}}}$, ${\widetilde{t}}_{{\rm{c}}}$). As a result, the *t*_c_ prior for the after-merger analysis (Extended Data Table 2) does not need to account for large trigger uncertainties and only needs to be large enough to capture expected posteriors, *p*(*t*_c_|**d**).

In the absence of external alerts, the independent DINGO-BNS search described earlier (Extended Data Fig. 1) can also be run as a pre-merger scan, searching for mergers around a freely chosen (but fixed) time, ${\widetilde{t}}_{{\rm{c}}}$, in the future. By continuously running this scan over the incoming data stream, alerts are triggered at roughly time ${\widetilde{t}}_{{\rm{c}}}$ before potential BNS mergers. Note that, in the absence of chirp mass predictions, we need to apply a conservative frequency range, [*f*_min_(*T*, ${\mathcal{M}}$_max_),*f*_max_(*−*$\widetilde{t}$_c_, ${\mathcal{M}}$_min_)], where ${\mathcal{M}}$_min*/*max_ are the prior bounds for ${\mathcal{M}}$.

#### EOS likelihood

A nuclear EOS implies a functional relationship between neutron star masses, *m*_*i*_, and tidal deformabilities, *λ*_*i*_. The likelihood, *p*(**d**|${\mathcal{E}}$), for a given EOS, ${\mathcal{E}}$, and data, **d**, can be computed by integrating the GW likelihood along the hyperplane defined by the EOS constraint, ${{\lambda }}_{i}={{\lambda }}_{i}^{{\mathcal{E}}}({m}_{i})$,

12 $$\begin{array}{l}p({\bf{d}}| {\mathcal{E}})=\int p({\bf{d}}| {\boldsymbol{\theta }})p({\boldsymbol{\theta }})\delta ({\lambda }_{i}-{\lambda }_{i}^{{\mathcal{E}}}({m}_{i})){\rm{d}}{\boldsymbol{\theta }}\\ \,=\int \int p({\bf{d}}| {m}_{i},{m}_{2},{\lambda }_{i}^{{\mathcal{E}}}({m}_{1}),{\lambda }_{2}^{{\mathcal{E}}}({m}_{2})){\rm{d}}{m}_{1}{\rm{d}}{m}_{2}\end{array}$$

Here *p*(**d**|*m*_1_,*m*_2_,*λ*_1_,*λ*_2_) is the Bayesian evidence of **d** conditional on (*m*_1_, *m*_2_, *λ*_1_, *λ*_2_). To calculate equation (12) using Monte Carlo integration, it is necessary to repeatedly evaluate the integrand, which is extremely expensive using traditional methods (for example, nested sampling).

With DINGO-BNS, there are two fast ways to evaluate the integrand, using either a conditional or a marginal network: (1) a marginal network, *q*(*m*_1_,*m*_2_,*λ*_1_,*λ*_2_|**d**), directly provides an unnormalized estimate of the conditional evidence, *p*(**d**|*m*_1_,*m*_2_,*λ*_1_,*λ*_2_) (sufficient for model comparison, but not subject to our usual accuracy guarantees); or (2) a conditional network, *q*(**θ**|**d**;*m*_1_,*m*_2_,*λ*_1_,*λ*_2_), provides the normalized conditional evidence via importance sampling (including accuracy guarantees). Option (1) allows 10^5^ evaluations per second, whereas option (2) only allows 10^3^, assuming 10^2^ weighted samples per evaluation.

**Extended Data Fig. 4:**
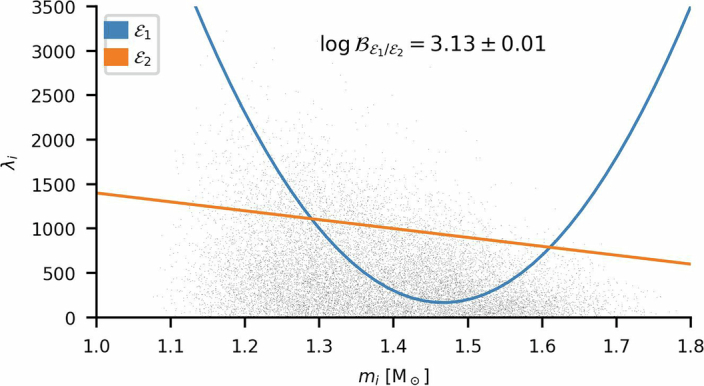
Neutron-star equation-of-state likelihood. Neutron-star EOS imply a functional relation *λ*_*i*_ = *λ*_*i*_(*m*_*i*_) between tidal parameters *λ*_*i*_ and component masses *m*_*i*_. The likelihood *p*(**d**|${\mathcal{E}}$) for an EOS ${\mathcal{E}}$ given the GW data **d** requires integrating the posterior *p*(**θ**|**d**) along the corresponding hyperplane. No posterior samples (gray) will be exactly on such hyperplanes (coloured lines), hence the standard Bayesian inference techniques are not directly applicable^87^. DINGO-BNS provides various possibilities to directly compute this quantity, enabling comparison of different EOS in terms of the Bayes factors ${\mathcal{B}}$.

By combining (1) and (2), we can achieve speed and accuracy, using the marginal network (1) to define a proposal distribution for Monte Carlo integration with the integrand from (2). Specifically, the density of the marginal network, *q*(*m*_1_,*m*_2_,*λ*_1_,*λ*_2_|**d**), is evaluated on an (*m*_1_,*m*_2_) grid with ${\lambda }_{i}={\lambda }_{i}^{{\mathcal{E}}}({m}_{i})$. This provides a discretized estimate of the integrand, *p*(**d**|*m*_1_, *m*_2_, ${\lambda }_{1}^{{\mathcal{E}}}$(*m*_1_), ${\lambda }_{1}^{{\mathcal{E}}}$(*m*_2_)), which we use as a proposal distribution for the integration in equation (12) when computing the integrand with the more accurate method (2). We tested this on GW170817 data using two polynomial EOS constraints, $\lambda ={\lambda }^{{\mathcal{E}}}(m)$ (Extended Data Fig. 4), finding good sample efficiencies of around 50%, small uncertainties, ${{\sigma }}_{\log p({\bf{d}}|{\mathcal{E}})}\approx 0.01$, and computation times of 1−3 s for the integral equation (12). Alternatively, the proposal could also be generated using a network, *q*(*m*_1_,*m*_2_|**d**), which additionally marginalizes over *λ*_*i*_. Finally, for a parametric EOS, a DINGO-BNS network could be conditioned on EOS parameters, allowing for direct EOS inference. This variety of approaches emphasizes the flexibility of SBI for EOS inference.

#### Related work

Machine learning for GW astronomy is an active area of research^55^. Several studies have explored machine-learning inference for black hole mergers^19,20,56–65^. There have also been applications specific to BNS inference (Extended Data Table 1). The GW-SkyLocator algorithm^66^ estimates the sky position using the SNR time series (similar to BAYESTAR), whereas Jim^33,35^ uses hardware acceleration and machine learning to speed up conventional samplers and achieve full inference in 21–33 min. The i-nessai framework^39^ achieves BNS inference in 24 min by combining normalizing flows using importance nested sampling. Ref. ^67^ explores pre-merger BNS detection and parameter estimation with normalizing flows, also reporting 1-s analysis times. However, it has not demonstrated accurate results on real data, and is subject to several other limitations. SBI has also been used for neutron star EOS inference from GWs^68^ and electromagnetic data^69^. Pre-merger localization with conventional techniques has been explored for ground-based third-generation detectors^70–72^ and for space-based detectors^73,74^.

### Experimental details

For our experiments, we trained DINGO-BNS networks using the hyperparameters and neural architecture^44,75^ from ref. ^19^, with a few modifications. The embedding network consisted of a sequence of 34 two-layer, fully-connected residual blocks with hidden dimensions of 2,048 (×8), 1,024 (×8), 512 (×6), 256 (×6) and 128 (×6) after the initial projection layer. Compared with ref. ^19^, this added ten new blocks, increasing the number of trainable parameters in this part of the embedding network from 17 million to 91 million. For the LVK experiments, we used a dataset with 3 × 10^7^ training samples and trained it for 200 epochs. For the Cosmic Explorer experiments, we used 6 × 10^7^ training samples and trained it for 100 epochs. Training took between 5 and 7 days on one H100 GPU. We used three detectors for LVK (LIGO-Hanford, LIGO-Livingston and Virgo) and two detectors for Cosmic Explorer (primary detector at the location of LIGO-Hanford, secondary detector at the location of LIGO-Livingston). The networks were trained with the priors displayed in Extended Data Table 2. The DINGO-BNS network marginalized over the phase of coalescence, *ϕ*_c_. During importance sampling, we reconstructed *ϕ*_c_ (ref. ^20^) (Extended Data Figs. 6a and 8) or used a phase-marginalized likelihood^76,77^ (in all other experiments). The phase reconstruction used here made the same assumptions as conventional phase marginalization^76,77^.

In the first experiment, we evaluated DINGO-BNS models on 200 simulated GW datasets, generated using a fixed GW signal with GW170817-like parameters and simulated LVK detector noise. We used noise PSDs from the second (O2) and third (O3) LVK, observing runs as well as LVK design sensitivity. For each noise level, we trained one pre-merger network (*f* ∈ [23,200] Hz) and one network for inference with the full signal, including the merger (*f* ∈ [23,1024]). The latter network was only used for after-merger inference because we found that separation into two networks improved the performance. The pre-merger network was trained with frequency masking, the masking bound, *f*_max_, being sampled in the range [28,200] Hz, enabling inference up to 60 s before the merger.

In the second experiment, we analysed 10^4^ simulated GW datasets, with GW signal parameters randomly sampled from the prior (Extended Data Table 2), the ${\mathcal{M}}$ prior reduced to the range [1.0,1.5] *M*_⊙_ and the *d*_L_ prior reweighted to a uniform distribution in the comoving volume, with design sensitivity noise PSDs. We again trained one pre-merger network (*f* ∈ [19.4,200] Hz) and one after-merger network (*f* ∈ [19.4, 1,024] Hz). The pre-merger network was trained with frequency masking with the masking bound, *f*_max_, sampled in the range [25,200] Hz, enabling inference up to 60 s before the merger for ${\mathcal{M}}$ ≤ 1.5 *M*_⊙_. Both networks were additionally trained with lower-frequency masking, with *f*_min_($\widetilde{{\mathcal{M}}}$) determined as explained above, ensuring an optimal frequency range for any chirp mass. Following ref. ^17^, we only considered events with SNR ≥ 12. Before each analysis, we performed a *t*_c_ scan by generating 2,500 samples for four time-shifted copies of the strain, followed by joint importance sampling of the combined results. Specifically, for a network with a *t*_c_ prior *U*(−${\tau }$, $\tau$), we applied the time shifts (−3$\tau$, −$\tau$, $\tau$, 3$\tau$), effectively increasing the prior to *U*(−4$\tau$, 4$\tau$). For the subsequent analysis, we time shifted the data such that the *t*_c_ posterior was fully covered by the prior.

**Extended Data Fig. 5:**
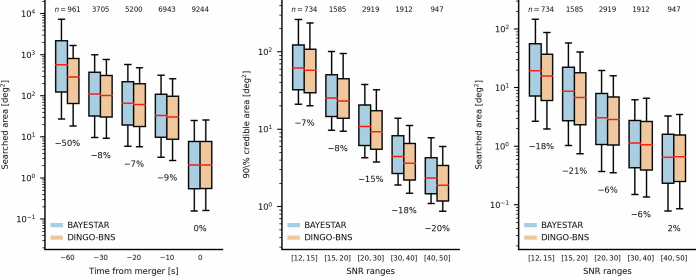
Localization comparison between BAYESTAR and DINGO-BNS. We evaluate the 90% credible area and the searched area. The boxplots display the medians (percentage changes indicated), quartiles and 10th/90th percentiles. The comparisons according to SNR are based only on results after the merger.

**Extended Data Fig. 6:**
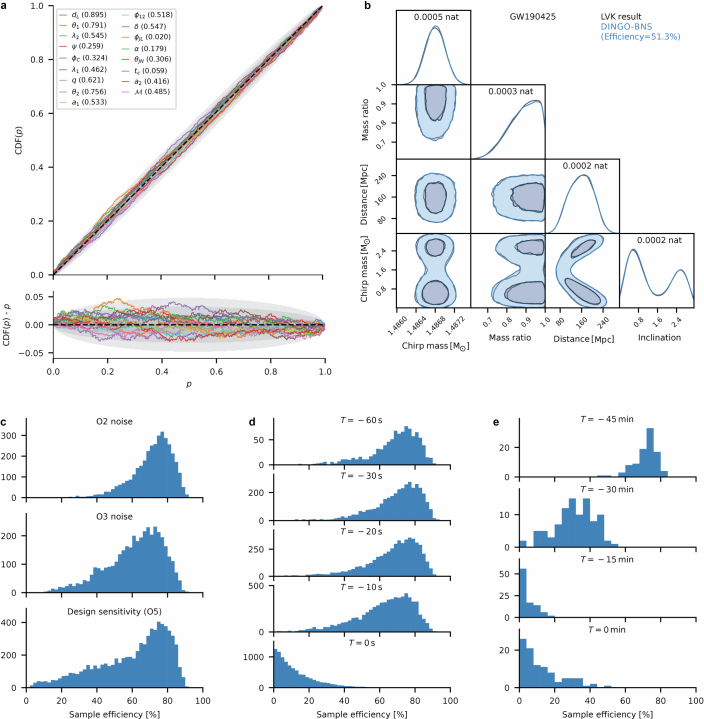
DINGO-BNS performance tests. (a) P-P plot for 1000 simulated GW datasets. For each simulation and parameter, we compute the percentile *p* of the injected parameter under the corresponding one-dimensional marginal posterior inferred with DINGO-BNS. The plot shows the cumulative distribution function (CDF) of *p*. For an unbiased sampler we expect CDF(*p*) = *p* within statistical uncertainties. Gray bands indicate confidence intervals corresponding to one, two and three standard deviations. The legend displays the *p*-value for each parameter, the combined *p*-value is 0.37. The GW datasets for this plot are generated with the settings from the after-merger analysis of the second experiment, except for the luminosity distance prior, which for this statistical test needs to be set to follow the uniform training prior (Extended Data Table 2). (b) Posterior for GW190425. 2D marginals are displayed in terms of 50% and 90% credible regions. For the 1D marginals, the deviation between the two results is quantified in terms of the Jensen–Shannon divergence (numbers on the diagonal). DINGO-BNS shows good agreement with the public LVK result^79^. (c)–(e) Sample efficiencies for DINGO-BNS, for (c) GW170817-like injections using different detector noise levels, (d) injections using LVK design sensitivity PSDs and (e) injections using CE PSDs. Panel b adapted from ref. ^79^ under a Creative Commons licence CC BY 4.0.

For each DINGO-BNS result, we generated a skymap using a kernel density estimator implemented by ligo.skymap^78^. For the sky localization comparison between DINGO-BNS and BAYESTAR, we ran BAYESTAR based on the GW signal template generated with the maximum likelihood parameters from the DINGO-BNS analysis. We noted that BAYESTAR was designed as a low-latency pipeline and typically run with (coarser) parameter estimates from search templates. Therefore, the reported BAYESTAR runs may deviate slightly from the realistic LVK setup. However, our results are consistent with those of ref. ^17^, which also had an approximately 30% precision improvement over BAYESTAR localization (using LVK search triggers). Both DINGO-BNS and ref. ^17^ performed full Bayesian BNS inference and should therefore have had identical localization improvements over BAYESTAR (assuming ideal accuracy, which for DINGO-BNS was validated with consistently high importance sampling efficiency). Differences to the localization comparison in ref. ^17^ are thus primarily attributed to different configurations for BAYESTAR and slightly different injection priors. Additional results for the localization comparison are shown in Extended Data Fig. 5. A probability–probability (P–P) plot for the after-merger analysis is shown in Extended Data Fig. 6a, which shows no significant bias for any parameter.

**Extended Data Fig. 7:**
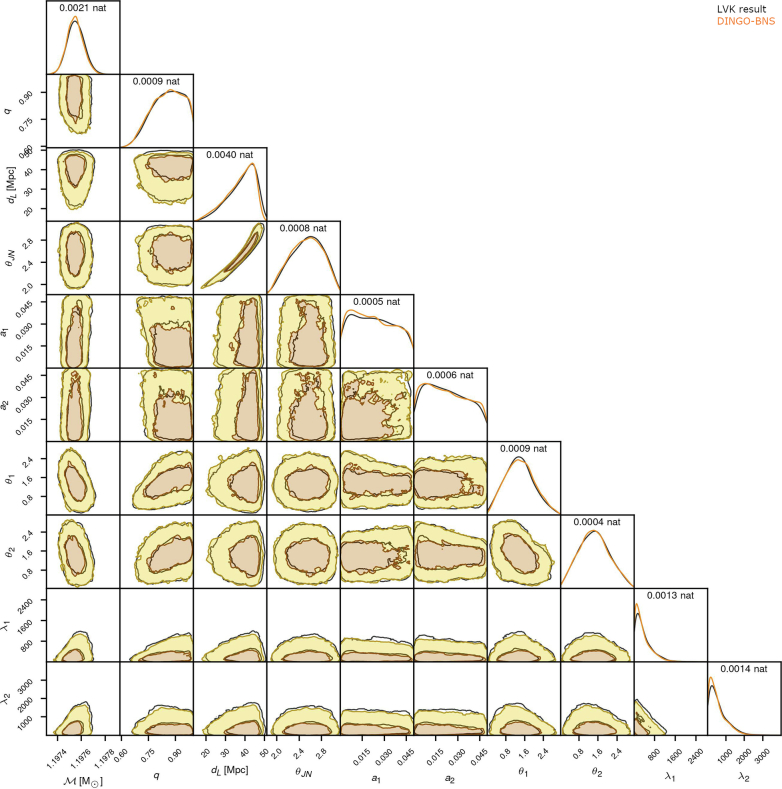
Extended posterior for GW170817. The corner plot compares the DINGO-BNS result from the main text (orange) with the LVK result (black)^5^. 2D marginals are displayed in terms of 50% and 90% credible regions. For the 1D marginals, the deviation between the two results is quantified in terms of the Jensen–Shannon divergence (numbers on the diagonal). For the definition of the parameters see Extended Data Table 2. The small deviations between the results could be a consequence of the marignalization over detector calibration uncertainties in the LVK result, which is not applied with DINGO-BNS.

In the third experiment, we reproduced the public LVK results for GW170817 (refs. ^1,5^) and GW190425 (ref. ^79^) with DINGO-BNS. We used the same priors and data settings as the LVK, but we did not marginalize over calibration uncertainty. The GW170817 after-merger analysis (see also Extended Data Fig. 7) was performed with a DINGO-BNS model conditioned on the sky position, {*α*,*β*}. Following the LVK analysis^5^, we used the localization *α* = 3.44616 rad and *δ* = *−*0.408084 rad from the electromagnetic counterpart AT 2017gfo (ref. ^8^) at inference. Note that the localization uncertainty (*σ*_*α*_ = 10^*−*6^ rad, *σ*_*δ*_ = 10^*−*6^ rad (ref. ^8^)) was negligible for GW parameter estimation, but such effects could, in principle, be integrated by convolving the conditional DINGO-BNS network with a distribution over {*α*,*β*}. We found good sample efficiencies for both events (10.8% for GW170817 and 51.3% for GW190425) and good agreement with the LVK results (Extended Data Fig. 6b). The LVK results used detector noise PSDs generated with BayesWave^36^, which were not available before the merger. For our pre-merger analysis of GW170817 in the main part (sample efficiency 78.9%), we thus used a PSD generated using the Welch method. The GW170817 signal overlapped with a loud glitch in the LIGO-Livingston detector^1^, and we used the glitch-subtracted data provided by the LVK in our analyses. Because such data would not be available before the merger, the pre-merger inference of BNS events overlapping with glitches would, in practice, also require fast glitch mitigation methods.

In the fourth experiment, we analysed simulated Cosmic Explorer data using the anticipated noise PSDs for the primary and secondary detectors. We trained a DINGO-BNS network for pre-merger inference using *f* ∈ [6,11] Hz, with the upper frequency masking bound, *f*_max_, sampled in the range [7,11] Hz. This supported a signal length of 4,096 s, with a pre-merger inference between 45 and 15 min before the merger. We injected signals with GW170817-like parameters for distance, masses and inclination to investigate how well a GW170817-like event could be localized in the Cosmic Explorer detector. We also trained a network on the full frequency range, [6, 1,024] Hz, for after-merger inference, with a reduced distance prior to control the SNR (Extended Data Table 2).

#### Sample efficiencies

We report sample efficiencies for all injection studies in Extended Data Fig. 6. The importance-sampled DINGO-BNS results are accurate, even with low efficiency, provided that a sufficient absolute number of effective samples can be generated. The efficiency nevertheless is a valuable diagnostic for assessing the performance of the trained inference networks.

In the LVK experiments, we found consistently high efficiencies, comparable to or greater than those reported for BBHs^20^. As a general trend, we observed that higher noise levels (Extended Data Fig. 6c) and earlier pre-merger times (Extended Data Fig. 6d) led to higher efficiencies. This is because low SNR events generally have broader posteriors, which are simpler to model for DINGO-BNS density estimators. Furthermore, the GW signal morphology is most complicated around the merger, making pre-merger inference much simpler than inference based on the full signal.

For Cosmic Explorer injections with GW170817-like parameters (Extended Data Fig. 6e), DINGO-BNS achieved extremely high efficiency for early pre-merger analyses, but the performance decreased substantially for later analysis times. This effect can again be attributed to the increase in SNR, which was *O*(10^3^) 15 min before the merger. Improving DINGO-BNS for such high SNR events will probably require improved density estimators^64^ that can better deal with tighter posteriors. When limiting the SNR by increasing the distance prior (Extended Data Table 2), we found good sample efficiencies for an after-merger Cosmic Explorer analysis that used the full 4,096-s-long signal (Extended Data Fig. 6e).

#### Inference times

The computational cost of inference with DINGO-BNS is dominated by: (1) neural-network forward passes to sample from the approximate posterior, ${\boldsymbol{\theta }} \sim q({\boldsymbol{\theta }}| {{\bf{d}}}_{\widetilde{{\mathcal{M}}}},\widetilde{{\mathcal{M}}})$; and (2) likelihood evaluations, *p*(**θ**|**d**), used for importance sampling. For 50,000 samples on an H100 GPU, (1) takes around 0.370 s and (2) takes around 0.190 s, resulting in an inference time of less than 0.6 s. The speed of the likelihood evaluations is enabled by using JAX waveform and likelihood implementations^33–35^, combined with the heterodyning and multibanding step that we also used to compress the data for the DINGO-BNS network. We extend the open-source implementations^34,35^ by combining NRTidalv1 (refs. ^32,80^) with IMRPhenomPv2 (refs. ^81–83^), as well as re-implementing the DINGO likelihood functions in JAX. The JAX functions are usually just-in-time compiled to run efficiently. The input dimension of the likelihood (determined by likelihood batch size and number of frequency bins) is fixed, enabling compilation ahead of time on random data of the correct input dimension. After compilation, a running DINGO-BNS script can perform any number of analyses without recompiling. Thus, we can leave the compilation time (18 s) out of the timing estimate for importance sampling. Compilation could further be transferred between separate DINGO-BNS runs using the persistent compilation cache in JAX, although we have not implemented this option. Likelihood evaluations can also be done without JAX, which takes less than 10 s on a single node with 64 CPUs for 50,000 samples. For the vast majority of DINGO-BNS analyses in this study, the sample efficiency was sufficiently high such that 50,000 samples corresponded to several thousands of effective samples after importance sampling, enabling full importance sampling inference in less than 1 s.

#### Additional sources of latency

The inference times quoted above assume that the data have already been provided to DINGO-BNS. In practice, there are various additional sources of latency.

**Extended Data Fig. 8:**
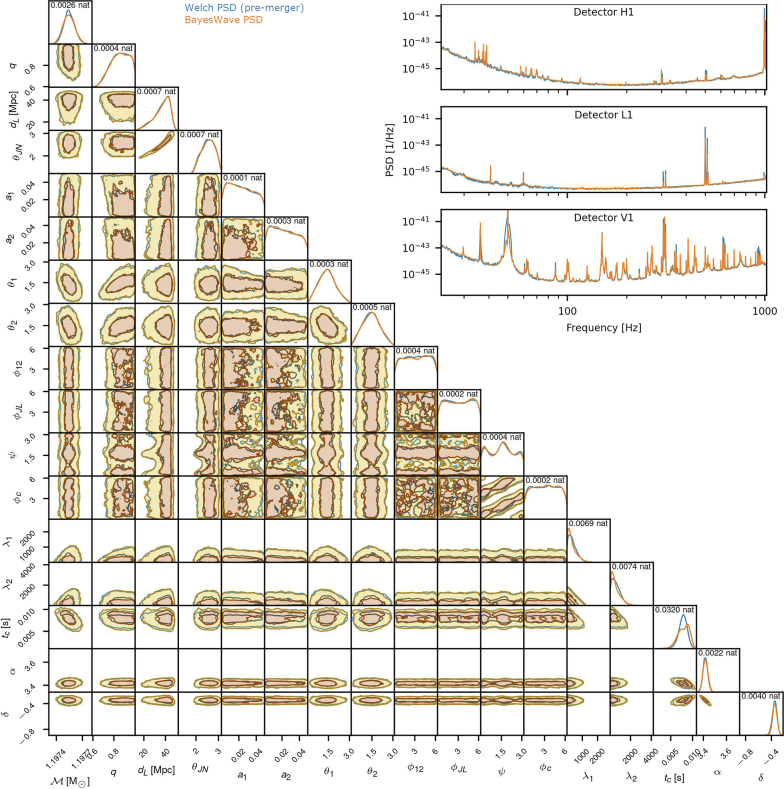
GW170817 posterior with different PSDs. The corner plot displays DINGO-BNS inference results using the BayesWave PSD (orange) from the LVK analysis and a Welch PSD (blue). 2D marginals are displayed in terms of 50% and 90% credible regions. For the 1D marginals, the deviation between the two results is quantified in terms of the Jensen–Shannon divergence (numbers on the diagonal). For the definition of the parameters see Extended Data Table 2. The Welch PSD is estimated based on strain data taken 1184 to 160 s before the BNS merger (using 128 segments of length eight seconds and subsequent upsampling to the desired resolution ∆*f* = 1/128 Hz), and is thus available in low latency for pre-merger analysis. The DINGO-BNS network for the Welch analysis is trained with a distribution of Welch PSDs covering the entire second LVK observing run, and tuned to the GW170817 PSDs via network conditioning^19^.

First, PSD estimation typically uses strain data taken during or after the merger. Obtaining low-latency PSDs represents a general challenge for low-latency analyses, encountered also in the field of GW searches^14,53^. Indeed, most PSDs used in this work would not be available in very low latency—the GW170817 and GW190425 PSDs from the LVK analyses^5,79^ use on-source noise estimation^36^ based on data during the merger, whereas LVK design sensitivity and Cosmic Explorer PSDs are not based on real detector noise, but rather reflect anticipated future configurations. These PSDs were chosen for comparability with existing studies. To test DINGO-BNS in a more realistic low-latency setting, we performed inference with PSDs estimated using Welch’s method^84^ and only data from before the merger (Extended Data Fig. 8). The resulting posterior for GW170817 only deviated slightly from the posterior obtained from the on-source PSDs. However, we note that, in general, such pre-merger Welch PSDs may be less reliable in the presence of non-stationary detector noise, which could lead to larger biases for parameter estimation.

Strain data can also be contaminated with instrumental or environmental glitches, which need to be removed for parameter estimation^85^. This adds additional latency for events that overlap with glitches, as noted above in the case of GW170817. Finally, data transfer between detectors and computing facilities add some additional latency. Using DINGO-BNS to its full potential will therefore require careful integration with low-latency pipelines^23^ and further acceleration of existing components.

#### PSD tuning

Although most of the networks used in this study were trained with only a single PSD per detector, in practice we would generally train DINGO-BNS with an entire distribution of PSDs to enable instant tuning to drifting detector noise^19^. (This is not relevant to tests involving, for example, design sensitivity noise.) Of the experiments in this study, only the pre-merger result of GW170817 and the result for the Welch PSD (Extended Data Fig. 8) were generated with a PSD-conditioned DINGO-BNS network. This network was trained with a PSD distribution covering the entire second LVK observing run (O2). Conditioning on the PSD makes the inference task more complicated and therefore leads to slightly reduced performance. For example, when repeating the first injection experiment (Extended Data Fig. 6c) with the PSD-conditioned DINGO-BNS network from above, the mean efficiency was reduced from 71% to 22%. Such networks can, in principle, also be trained before the start of an observing run, by training with a synthetic dataset designed to reflect the expected noise PSDs^86^.

## Online content

Any methods, additional references, Nature Portfolio reporting summaries, source data, extended data, supplementary information, acknowledgements, peer review information; details of author contributions and competing interests; and statements of data and code availability are available at 10.1038/s41586-025-08593-z.

## Figures and Tables

**Extended Data Table 1:**
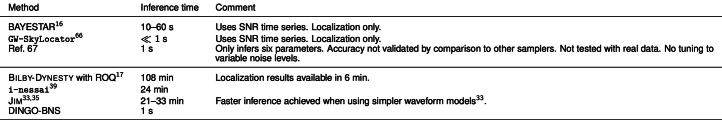
Comparison of fast methods for BNS inference

The four methods in the lower block all perform full parameter estimation and their accuracy on real data has been validated by comparison against established samplers.

**Extended Data Table 2:**
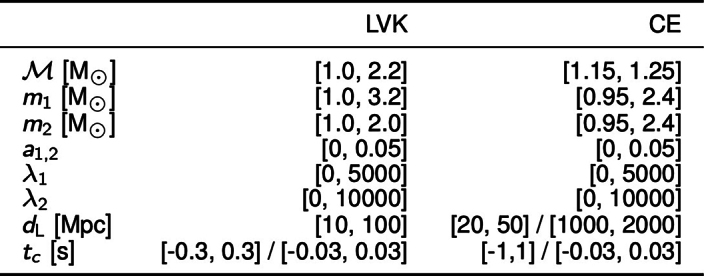
Training priors

Parameters include chirp mass ${\mathcal{M}}$, component masses *m*_1,2_, spin magnitudes *a*_1,2_, tidal deformabilities *λ*_1,2_, luminosity distance *d*_L_ and merger time *t*_c_. All priors are uniform, except for chirp mass, which is sampled uniform in component masses. At inference, *d*_L_ can be reweighted to the standard prior (uniform in comoving volume). For *t*_c_ we use a broader prior for pre-merger inference than for full inference (separated by “/” symbol) to account for higher uncertainties. We have verified that the *t*_c_ priors are sufficiently broad for the data analyzed here, although broader priors may be required for analyzing events with lower SNR or earlier pre-merger times. LVK priors are chosen to cover expected LVK BNS detections. CE priors for ${\mathcal{M}}$ and *d*_L_ are reduced compared to LVK to decrease the computational cost of training. Priors for parameters not displayed here (sky position (*α*,*δ*), spin angles (*θ*_1_,*θ*_2_,*Φ*_12_,*Φ*_*JL*_), inclination *θ*_*JN*_, polarization angle *ψ* and reference phase *Φ*_*c*_) are standard. The prior for mass ratio *q* is determined via the priors for ${\mathcal{M}}$ and *m*_1,2_.

## Data Availability

Public LVK data is available at https://gwosc.org/events/GW170817/ for GW170817 and at https://dcc.ligo.org/LIGO-T1900685/public for GW190425.

## Code availability

The code for DINGO-BNS is publicly available as an extension of the DINGO python package at https://github.com/dingo-gw/dingo. We provide a demo at https://github.com/dingo-gw/binary-neutron-star-demo and a trained network via Zenodo at https://zenodo.org/records/13321251 (ref. 88).
